# SP1-Induced Upregulation of LncRNA AFAP1-AS1 Promotes Tumor Progression in Triple-Negative Breast Cancer by Regulating mTOR Pathway

**DOI:** 10.3390/ijms241713401

**Published:** 2023-08-29

**Authors:** Fangyuan Li, Daheng Xian, Junying Huang, Longzhu Nie, Ting Xie, Qiang Sun, Xiaohui Zhang, Yidong Zhou

**Affiliations:** 1 Clinical Biobank, Peking Union Medical College Hospital, Chinese Academy of Medical Sciences (CAMS) and Peking Union Medical College, Beijing 100730, China; doris_lfy@163.com (F.L.); xieting@pumch.cn (T.X.); 2 Department of Breast Surgery, Peking Union Medical College Hospital, Chinese Academy of Medical Sciences (CAMS) and Peking Union Medical College, Beijing 100032, China; joesin1996@gmail.com (D.X.); hjy919447268@163.com (J.H.); nielongzhu0606@163.com (L.N.); sunqiangpumch@sina.com (Q.S.)

**Keywords:** SP1, LncRNA, AFAP1-AS1, mTOR

## Abstract

The long non-coding RNA (lncRNA) actin fiber-associated protein-1 antisense RNA 1 (AFAP1-AS1) exerted oncogenic activity in triple-negative breast cancer (TNBC). We designed this study and conducted it to investigate the upstream regulation mechanism of AFAP1-AS1 in TNBC tumorigenesis. In this work, we proved the localization of AFAP1-AS1 in the cytoplasm. We elucidated the mechanism by which the transcription factor specificity protein 1 (SP1) modulated AFAP1-AS1 in TNBC progression, which has yet to be thoroughly studied. Dual luciferase reporter assay and chromatin immunoprecipitation (ChIP) assay revealed a strong affinity of SP1 toward the promoter regions P3 of AFAP1-AS1, proving the gene expression regulation of AFAP1-AS1 via SP1 in TNBC. Additionally, SP1 could facilitate the tumorigenesis of TNBC cells in vitro and in vivo by regulating the AFAP1-AS1 expression. Furthermore, silenced AFAP1-AS1 suppressed the expression of genes in the mTOR pathway, such as eukaryotic translation initiation factor 4B (EIF4B), mitogen-activated protein kinase-associated protein 1 (MAPKAP1), SEH1-like nucleoporin (SEH1L), serum/glucocorticoid regulated kinase 1 (SGK1), and its target NEDD4-like E3 ubiquitin protein ligase (NEDD4L), and promoted the gene expression of s-phase kinase-associated protein 2 (SKP2). Overall, this study emphasized the oncogenic role of SP1 and AFAP1-AS1 in TNBC and illustrated the AFAP1-AS1 upstream interaction with SP1 and the downstream modulatory of mTOR signaling, thus offering insights into the tumorigenesis mechanism in TNBC.

## 1. Introduction

Triple-negative breast cancer (TNBC), a biological and clinically heterogeneous disease, accounts for 15 to 20% of all breast cancers (BC) and is more common in premenopausal women or those with breast cancer gene 1 (BRCA1) gene mutation [1]. It has been defined by the lack of expression (<1%) of estrogen receptor (ER), progesterone receptor (PR), and human epidermal growth factor receptor (HER2), and has a poorer prognosis and worst 5-year relative survival rate compared to other BC subtypes [2,3]. The therapeutic strategies development in TNBC is limited, and conventional chemotherapy is the mainstay of treatment because of the lack of well-accepted reliable biological targets or biomarkers. So, researchers paid much attention to TNBC, and the understanding of the molecular and genetic background of TNBC has gradually improved in the past few years [4,5,6].

Ninety percent of the human genome comprises non-coding genes [7]. Long non-coding RNAs (lncRNA) are transcripts longer than 200 bp and prove to function as cancer regulators. Dysregulation of lncRNA has been found in plenty of cancers and is associated with tumor progression, metastasis, or drug resistance [8,9,10]. In TNBC, lncRNAs play essential roles in its multistep tumorigenesis process [11,12]. For example, as the most studied molecular mechanism, lncRNAs function in competing endogenous RNA (ceRNA) mechanisms, thereby affecting the miRNA-target gene regulation [13]. Moreover, lncRNAs can assemble with mRNAs and increase their stability. Some lncRNAs serve as scaffolds to assemble molecular components or to function to interact with transcription factors or enzymes to *cis*- or *trans*-regulate the target gene expression [14].

LncRNA actin filament-associated protein 1 antisense RNA 1 (AFAP1-AS1, NC_000004.12) was transcribed from *AFAP1* gene located in chromosome 4. It has mainly been regarded as oncogenic lncRNA and is associated with tumor progression [15]. This oncogenic effect functions through the modulation of expression in cancer-related miRNAs. In TNBC cells, overexpressed AFAP1-AS1 competitively bound to miR-145 [16] and miR-2110 [17] and promote the expression of activating transcription factor 6 (ATF6)/MTH1 [16] and transcription factor specificity protein 1 (SP1) [17], thereby increasing cell proliferation and invasion by function in ceRNA mechanisms. Furthermore, AFAP1-AS1 has been shown to affect TNBC progression via activating epithelial-mesenchymal transition (EMT) of TNBC cells by increasing the Wnt/β-catenin signaling and C-myc [18]. Moreover, those studies’ BALB/c nude mice models verified AFAP1-AS1 oncogenic roles. However, the mechanism of how AFAP1-AS1 was modulated in regulating TNBC remains unclear.

In this study, we conducted and performed experiments to investigate the upstream regulation mechanism of AFAP1-AS1 in TNBC tumorigenesis. Mechanically, we proved the binding and expression regulation of SP1 toward the AFAP1-AS1, thereby facilitating the tumorigenesis of TNBC cells in vitro and in vivo. Moreover, we found that silenced AFAP1-AS1 could suppress the expression of 411 and 273 genes in MDA-MB-231 and MDA-MB-468 cells, especially in mechanistic/mammalian target of rapamycin (mTOR) signaling. In general, this study demonstrated the upstream regulation of AFAP1-AS1 and its downstream modulatory of mTOR signaling, thereby providing a supplementary mechanism of AFAP1-AS1 toward the tumorigenesis mechanism in TNBC.

## 2. Results

### 2.1. SP1 Silencing Decreases the Expression of lncRNA AFAP1-AS1

Studies have shown that some key transcription factors and multiple epigenetic regulators may lead to dysregulated expression of lncRNAs in human cancers. The online prediction tool, JASPAR (http://jaspar.genereg.net/, accessed on 13 July 2022), was used to explore the potential transcription factors at the promoter of lncRNA AFAP1-AS1 (hg38_knownGene_ENST00000608442.2, range = chr4:7752077–7754076). The results revealed that SP1 and YY1 transcription factor (YY1) had potential binding sites at the promoter regions of lncRNA AFAP1-AS1 (Table S2). As we have studied before, lncRNA AFAP1-AS1 was upregulated in TNBC tissues compared to the normal tissues, and SP1 and YY1 were also overexpressed in TNBC [17,19]. Therefore, SP1 (or YY1) may directly interact with the promoter region of lncRNA AFAP1-AS1 as upstream transcription factors, thereby regulating the expression of lncRNA AFAP1-AS1 and promoting or inhibiting tumor progression. 

**Figure 1:**
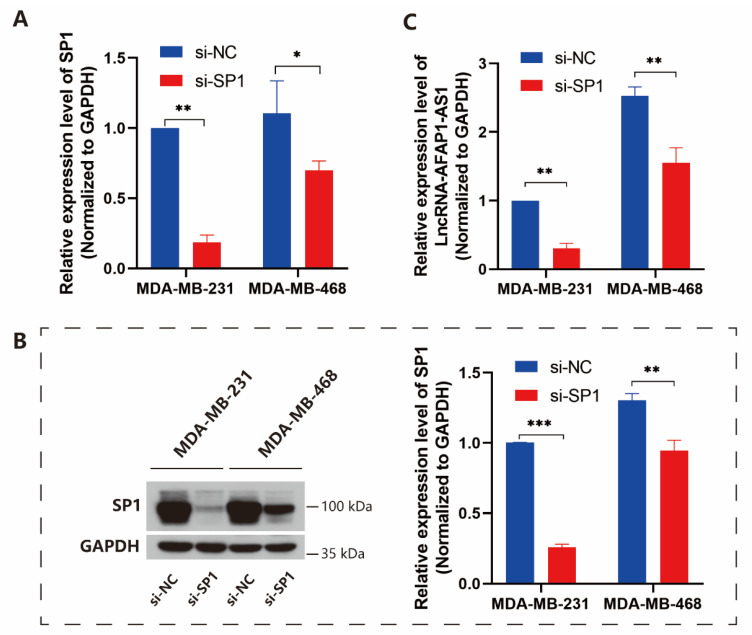
SP1 increases the expression of lncRNA AFAP1-AS1. (**A**) Q-RT-PCR and (**B**) western blot assays confirmed the silencing efficiency of SP1 by siRNA; (**C**) lncRNA AFAP1-AS1 expression significantly decreased in MDA-MB-231 and MDA-MB-468 cells after SP1 silencing. Unpaired Student’s *t*-tests were used for the statistical analyses. * *p* < 0.05; ** *p* < 0.01; *** *p* < 0.005.

In order to identify whether SP1 or YY1 regulated lncRNA AFAP1-AS1 expression, we first silenced SP1 and YY1 in MDA-MB-231 and MDA-MB-468 cells using siRNAs. Q-RT-PCR and western blot assays confirmed the silencing efficiency. The results showed that after the silence of SP1 and YY1, the si-SP1 and si-YY1 exhibited superior silencing efficiency in MDA-MB-231 and MDA-MB-468 cells, confirming in both mRNA and protein levels (Figure 1A,B and Figure S1A,B). Then, Q-RT-PCR evaluated the mRNA expression of the lncRNA AFAP1-AS1, and the results showed that lncRNA expression decreased in MDA-MB-231 and MDA-MB-468 cells after SP1 silencing (Figure 1C). When YY1 was silenced, lncRNA expression in MDA-MB-231 cells decreased as expected. In contrast, the expression abnormally increased in MDA-MB-468 cells (Figure S1C). So, SP1 was selected in subsequent studies.

### 2.2. SP1 Binds to the Promoter Region of lncRNA AFAP1-AS1

**Figure 2:**
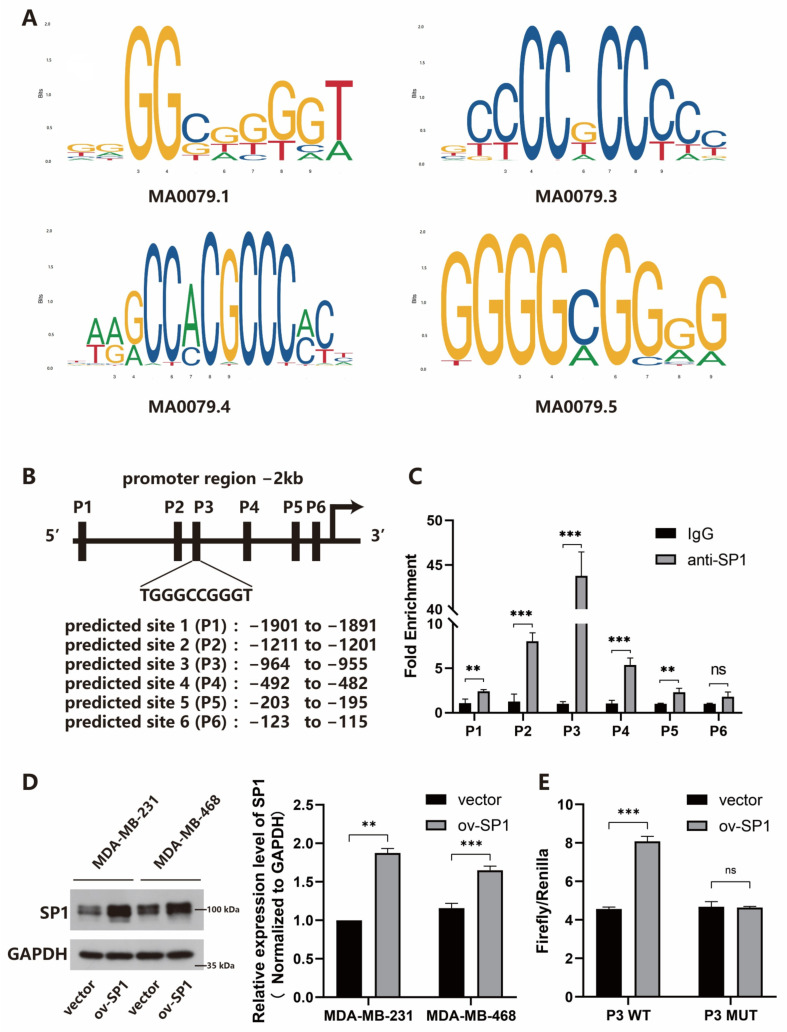
SP1 transcriptionally activates lncRNA AFAP1-AS1 in TNBC cells. (**A**) motif plot for SP1; (**B**) predicted SP1 binding sites in lncRNA AFAP1-AS1 promoter sequence by JASPAR; (**C**) ChIP assay confirmed the binding of SP1 to lncRNA AFAP1-AS1 promoter region at P3; (**D**) western blot confirmed the overexpression of SP1 by ov-SP1 vector; (**E**) dual-luciferase assay determined the binding site P3. Unpaired Student’s *t*-tests were used for the statistical analyses. ** *p* < 0.01; *** *p* < 0.005; ns, not significant.

ChIP assay results showed that the enrichment of lncRNA AFAP1-AS1 promoter in the complexes conjugated with anti-SP1, indicating that SP1 bound with lncRNA AFAP1-AS1 promoter, and P3 region was the most enriched promoter (Figure 2A–C). Western blot evaluated the overexpression of SP1 conducted by the ov-SP1 vector. The results confirmed that the SP1 protein upregulated both in MDA-MB-231 and MDA-MB-468 cells after the transfection of the SP1 overexpression vector (ov-SP1) (Figure 2D). SP1 overexpression increased the luciferase activity of the lncRNA AFAP1-AS1-WT promoter sequence at the P3 region (1037–1046), while the luciferase activity of the P3 mutant sequence remained unaffected (Figure 2E). The data above indicated that SP1 bound to the P3 promoter region to promote lncRNA AFAP1-AS1 expression.

### 2.3. SP1 Increases the Expression of lncRNA AFAP1-AS1 and Promotes the lncRNA AFAP1-AS1-Mediated Proliferation and Migration of TNBC Cells

**Figure 3:**
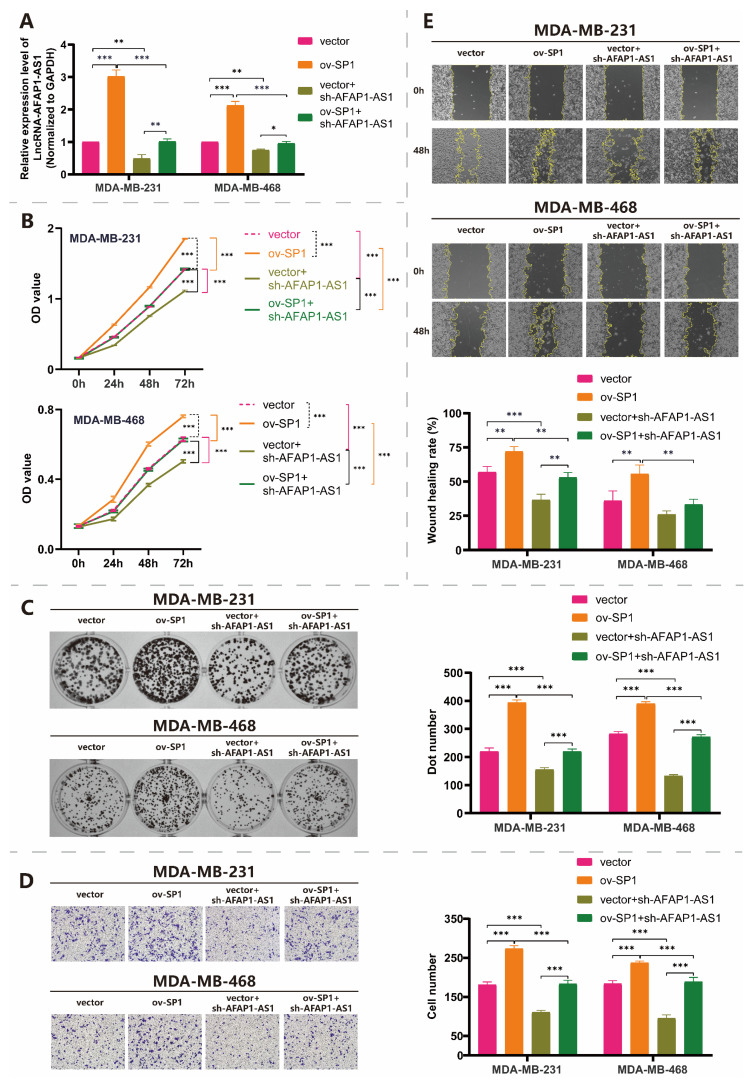
SP1 promotes the lncRNA AFAP1-AS1-mediated proliferation and migration of TNBC cells. (**A**) Q-RT-PCR assay confirmed that SP1 overexpression rescued the lncRNA AFAP1-AS1 expression caused by lncRNA AFAP1-AS1 silencing. (**B**) CCK-8 assay (the statistical analyses and *p*-value shown here were analyzed in 72 h group) and (**C**) colony formation assay evaluated the promotion of lncRNA AFAP1-AS1 mediated proliferation of TNBC cells by SP1 overexpression; (**D**) Transwell assay (100X) and (**E**) wound healing assay in 48 h evaluated the promotion of lncRNA AFAP1-AS1 mediated migration of TNBC cells by SP1 overexpression. Two-way ANOVA was used for CCK-8 analyses. One-way ANOVA was used for the other statistical analyses. * *p* < 0.05; ** *p* < 0.01; *** *p* < 0.005.

In order to probe the SP1 effect on cell behaviors mediated by lncRNA AFAP1-AS1, we conducted the gain and loss function assays by the overexpression of SP1 (ov-SP1) and silencing of lncRNA AFAP1-AS1 (sh-AFAP1-AS1). First, Q-RT-PCR confirmed the transfection efficiency, and the results showed that lncRNA AFAP1-AS1 expression significantly increased after the overexpression of SP1 by the ov-SP1 vector (group ov-SP1 vs. group vector). This overexpression rescued the lncRNA AFAP1-AS1 expression inhibition caused by lncRNA AFAP1-AS1 silencing (group vector vs. group vector + sh-AFAP1-AS1; group vector + sh-AFAP1-AS1 vs. group ov-SP1 + sh-AFAP1-AS1) in both MDA-MB-231 and MDA-MB-468 cells (Figure 3A). Furthermore, CCK-8 and colony formation assays showed that SP1 overexpression resulted in activated cell viability and rescued the lncRNA AFAP1-AS1 mediated proliferation inhibition caused by sh-AFAP1-AS1 (Figure 3B,C). SP1 also promoted the lncRNA AFAP1-AS1 mediated migration inhibition of TNBC cells by Transwell (Figure 3D) and wound healing assay (Figure 3E).

### 2.4. SP1 Facilitates the Tumorigenesis of TNBC Cells In Vivo

**Figure 4:**
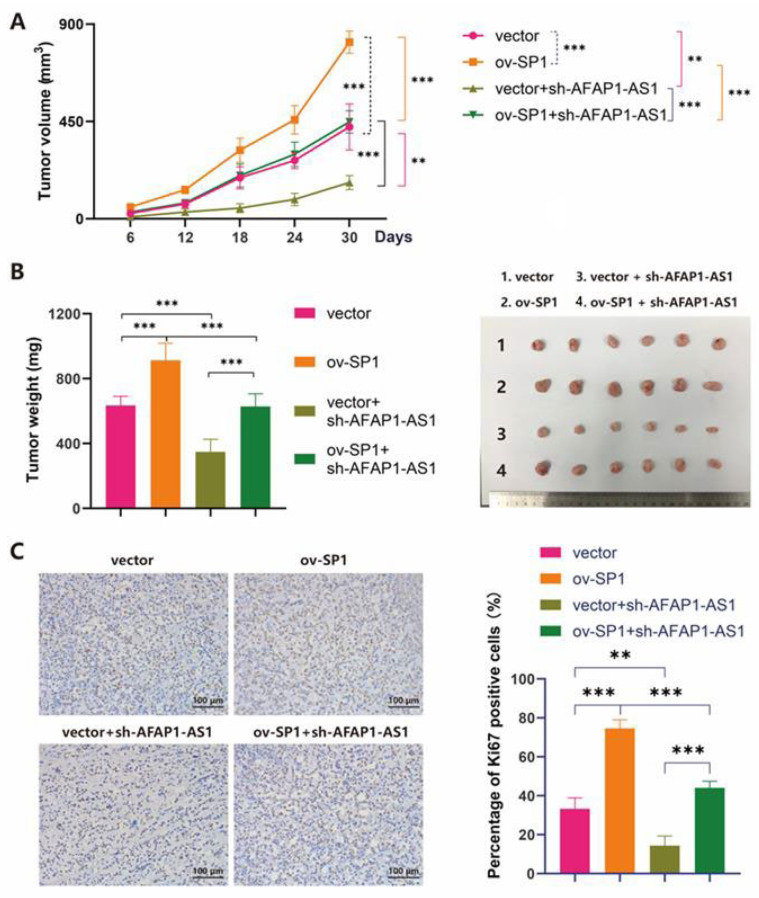
SP1 promotes the tumorigenesis of TNBC cells in vivo. (**A**) Tumor volume (**B**) tumor weight and tissues in nude mice treated with empty vector, ov-SP1, vector + sh-AFAP1-AS1, or ov-SP1 + sh-AFAP1-AS1(the statistical analyses and *p*-value shown here were analyzed in 30 Days). (**C**) Immunohistochemical staining of Ki67 in tumor tissues of mice. Two-way ANOVA was used for tumor volume growth analyses. Moreover, one-way ANOVA was used for the other statistical analyses. ** *p* < 0.01; *** *p* < 0.005.

We then performed xenograft tumors in nude mice to evaluate the effect of SP1 on the tumorigenesis of MDA-MB-231 cells in vivo. SP1 overexpression promoted tumor growth (group ov-SP1 vs. group vector). In contrast, the silencing of AFAP1-AS1 suppressed tumor growth (group vector + sh-AFAP1-AS1 vs. group vector), which could be reversed by overexpression of SP1 (group vector + sh-AFAP1-AS1 vs. group ov-SP1 + sh-AFAP1-AS1) (Figure 4A,B). Immunohistochemistry staining was then utilized to assess the protein expression of Ki67. The percentage of Ki67 positive cells (%) in the ov-SP1 group (74.6%) was significantly increased compared to the vector control group (33.2%). In contrast, the percentage of Ki67 positive cells (%) decreased in the sh-AFAP1-AS1 group (14.4%) compared with the vector group (33.2%). Meanwhile, the percentage of Ki67 positive cells (%) also increased in the ov-SP1 + sh-AFAP1-AS1 group (44.0%) compared with the vector + sh-AFAP1-AS1 group (14.4%) (Figure 4C). These findings demonstrated that overexpression of SP1 could reverse tumor growth at a slower speed caused by sh-AFA1-AS1 in vivo.

### 2.5. LncRNA AFAP1-AS1 Modulates Transcriptome Landscape

Our group previously proved the activation of overexpressed lncRNA AFAP1-AS1 towards TNBC tumorigenesis [17]. We next attempted to describe its detailed molecular mechanisms at a transcriptome level. We performed GeneChip^®^ PrimeView™ Human Gene Expression Array after the knockdown of lncRNA AFAP1-AS1 (sh-AFAP1-AS1) in MDA-MB-231 and MDA-MB-468 cells. 

**Figure 5:**
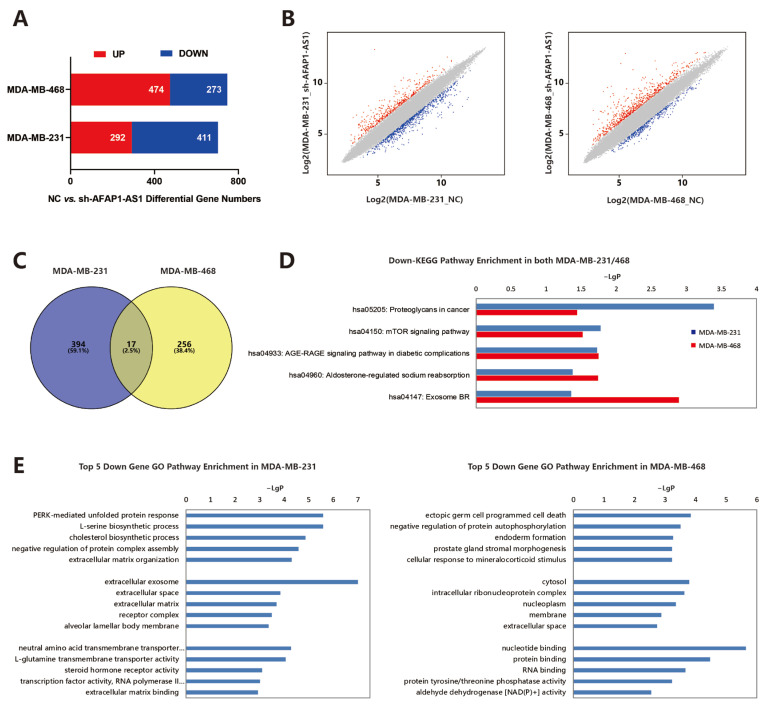
lncRNA AFAP1-AS1 modulates the gene expression landscape. (**A**) The number and (**B**) the scatter chart of changed genes after the knockdown of lncRNA AFAP1-AS1. (**C**) The overlapping of down-regulated genes between MDA-MB-231 and MDA-MB-468 cells. (**D**) The down-enriched KEGG pathways analysis in both MDA-MB-231 and MDA-MB-468 cells. (**E**) The top 5 down GO enrichment analysis in MDA-MB-231 and MDA-MB-468 cells.

The genes having a fold change in expression > 2 and *p*-value ≤ 0.05 were considered to be significantly changed. We found 292 up-regulated and 411 down-regulated mRNAs in MDA-MB-231 cells and 474 up-regulated and 273 down-regulated mRNAs in MDA-MB-468 cells after the knockdown of lncRNA AFAP1-AS1 (Figure 5A,B). 

The intersection of the down-regulated genes between MDA-MB-231 and MDA-MB-468 cells contained 17 genes, listed in Table S3 (Figure 5C). These 17 genes were involved in 44 KEGG pathways. For example, eukaryotic translation initiation factor 4B (EIF4B) and serum/glucocorticoid-regulated kinase 1 (SGK1) were both related to the mTOR signaling pathway (KEGG hsa04150).

In another aspect, KEGG pathway analysis indicated that five KEGG pathways enriched in both MDA-MB-231 and MDA-MB-468 cell lines (Figure 5D). The mTOR signaling pathway (KEGG hsa04150) and aldosterone-regulated sodium reabsorption (KEGG hsa04960) appeared to be significantly affected by the knockdown of lncRNA AFAP1-AS1.

Moreover, gene ontology (GO) analysis also revealed that these commonly predicted genes were associated with cancers (Figure 5E).

### 2.6. LncRNA AFAP1-AS1 Modulates the Gene Expression of the mTOR Pathway

**Figure 6:**
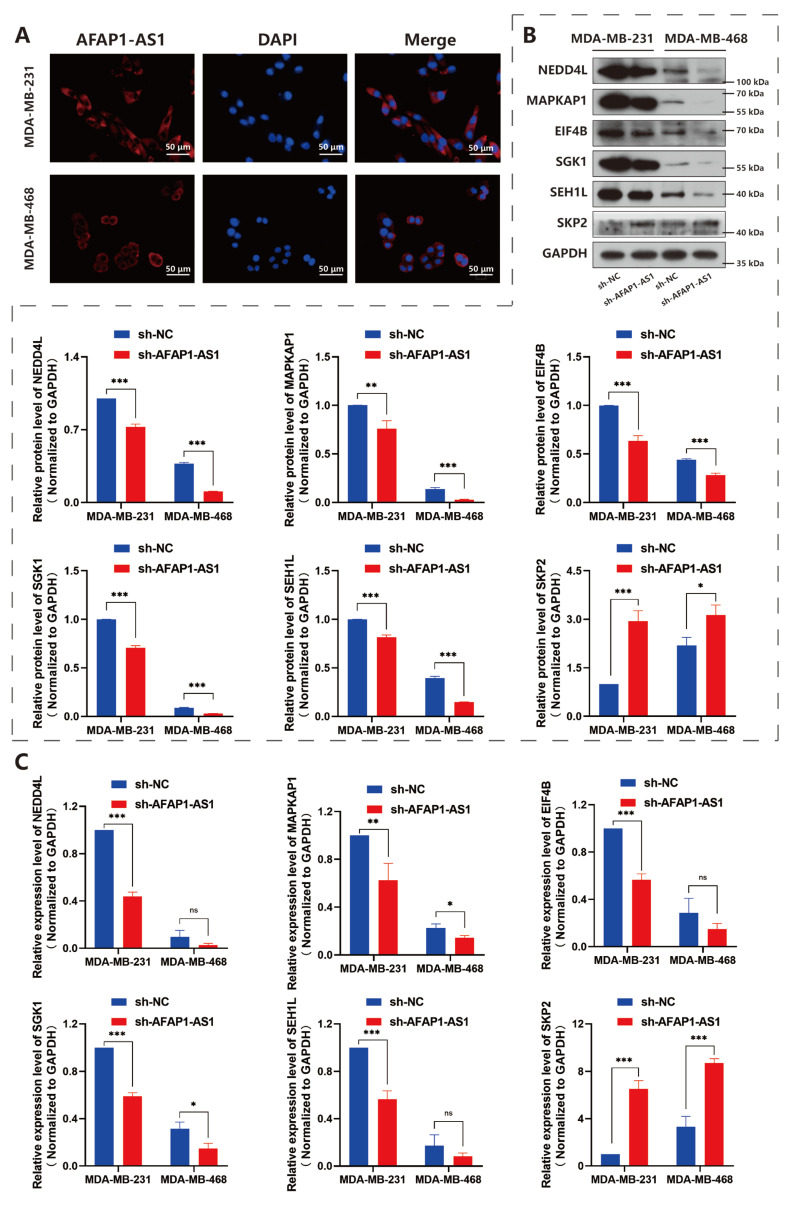
LncRNA AFAP1-AS1 modulates the gene expression of the mTOR pathway. (**A**) FISH analysis indicated the localization of most lncRNA AFAP1-AS1 in the cytoplasm. (**B**) Western blot and (**C**) Q-RT-PCR evaluated mTOR pathway genes’ protein and mRNA expression. Unpaired Student’s *t*-tests were used for the statistical analyses. * *p* < 0.05; ** *p* < 0.01; *** *p* < 0.005; ns, not significant.

Fluorescence in situ hybridization (FISH) analysis indicated that the majority of lncRNA AFAP1-AS1 was located in the cytoplasm (Figure 6A). Once located in the cytoplasm, lncRNA mostly *trans*-regulated gene expression at the post-transcriptional level, such as regulating mRNA translation and degradation, or participates in the regulation of intracellular signal pathways through protein binding, sponging miRNA, or base pairing with target RNAs [14].

Realizing that the mTOR signaling pathway (KEGG hsa04150) and aldosterone-regulated sodium reabsorption (KEGG hsa04960) significantly changed after AFAP1-AS1 knockdown, we performed the literature retrieval, and the results found that the mTOR signaling pathway had a closer relationship with TNBC tumorigenesis [20,21,22].

Then, we evaluated some gene expression in the mTOR pathway according to the gene expression of GeneChip^®^ results. The expression of mammalian stress-activated map kinase-interacting protein 1 (mSin1, also known as MAPKAP1), EIF4B, SGK1, and SEH1-like nucleoporin (SEH1L) positively regulated, while s-phase kinase-associated protein 2 (SKP2) negatively regulated by AFAP1-AS1. NEDD4-like E3 ubiquitin protein ligase (NEDD4L) proved to be phosphorylated by SGK1 [23] and was shown to be positively regulated by AFAP1-AS1 (Figure 6B,C). The results confirmed the downstream modulatory of AFAP1-AS1 toward mTOR signaling.

## 3. Discussion

AFAP1-AS1 was upregulated in almost all kinds of malignant tissues, such as lung cancer [24,25], breast cancer [16,26], and osteosarcoma [27]. Its upregulation had a significant impact on increasing malignancy and decreasing the survival of patients [28]. Primarily, overexpression of AFAP1-AS1 was found in TNBC tissues and cells, exerting oncogenic activity, and was associated with poor survival in TNBC patients [29]. Several researchers have focused on the downstream molecular mechanism of AFAP1-AS1 toward TNBC, primarily through its function as a sponge of cancer-related miRNAs. In comparison, scientists lightly discovered the upstream regulation of AFAP1-AS1. Only research in nasopharyngeal carcinoma had described that SP1 induced AFAP1-AS1, which could promote nasopharyngeal carcinoma progression by miR-497-5p/CUGBP Elav-like family member 1 (CELF1) [30]. The upstream mechanism of AFAP1-AS1 in TNBC tumor progression remains unclear. By utilizing the publicly available software JASPAR (http://jaspar.genereg.net/, accessed on 13 July 2022), we first searched the potential transcriptional binding motifs in the promoter of AFPA1-AS1. SP1 was the potential upstream regulator of AFAP1-AS1. SP1 belongs to the SP transcription factor family and functions in the transcriptions of many genes, which are essential for cancer development [31]. SP1 suppression may be a potential intervention strategy for inhibiting the migration of breast cancer cells [32] and slowing down the progression of TNBC [33]. Some studies have mentioned the activation of SP1 toward lncRNAs. For example, SP1 activated LINC00659 in gastric cancer, promoting tumor progression by miR-370/aquaporin 3 (AQP3) axis [34]. SP1 upregulated lncRNA TUG1 and promoted the tumorigenesis of colorectal cancer cells by TUG1/miR-421/lysine demethylase 2A (KDM2A)/ERK axis [35]. Moreover, SP1 bound directly to the promoter regions of lncRNA PANDAR [36] and lncRNA SPRY4-IT1 [37] and activated their transcription. The present study found SP1 to be an upstream regulator of AFAP1-AS1, promoting AFAP1-AS1 transcription by targeting its promoter region P3. Then, we evaluated SP1′s oncogenic activity in lncRNA AFAP1-AS1 mediated proliferation and migration of TNBC cells and its promotion on tumorigenesis in vivo assays. We proved the presumption in the previously reported study that AFAP1-AS1 reduced the inhibitory effect of miR-2110 on SP1 [17], and the elevated SP1 bound to the AFAP1-AS1 promoter region and activated its transcription, in turn, as the feedback system.

The downstream molecular mechanism of AFAP1-AS1 affection toward TNBC has shown to affect several aspects of breast carcinogenesis, and most of them were exerted through modulation of expression of cancer-related miRNAs. For example, AFAP1-AS1 bound miR-497-5p and regulated the expression of septin 2 (SEPT2), resulting in the modulation of progression in breast cancer cells [38]. miR-145 was another target of AFAP1-AS1, and the AFAP1-AS1/miR-145/MTH1 axis regulated the proliferation and invasiveness of TNBC cells [16]. AFAPA-AS1/miR-2110/SP1 axis contributed to the TNBC progression [17]. Except for ceRNA functions, AFAP1-AS1 could also indirectly influence the activity of some cancer-related pathways, such as Wnt/β-catenin. Overexpressed AFAP1-AS1 activated the Wnt/β-catenin pathway to promote tumorigenesis and cell invasion by regulating the expression of C-myc and EMT molecules in TNBC cells [18]. In this study, AFAP1-AS1 changed the landscape of gene mRNA expression and activated the mTOR pathway.

mTOR, a downstream effector of the phosphoinositide-3-kinase (PI3K)/AKT kinase (AKT) pathway, was a serine-threonine kinase that interacted with several proteins to form two distinct mTOR complexes, mTORC1 and mTORC2 [21]. mTORC1 recruited and phosphorylated eukaryotic translation initiation factor 4E binding protein 1 (EIF4EBP1) and ribosomal protein S6 kinase (S6K), and its activation led to downstream effects, such as protein synthesis promotion [39,40,41]. mTORC2 activated protein kinase C-α (PKC-α)/AKT and regulated the actin cytoskeleton [42]. In brief, these two complexes performed different functions: mTORC1 regulated cell growth and metabolism, while mTORC2 primarily controlled cell survival and proliferation [43]. Deregulation of the mTOR pathway (such as the overexpression of S6K1 and EIF4EBP1) is often found in human cancers (such as breast cancer) and promotes cell proliferation [20,22]. Except for the classic cellular pathways upstream of mTOR (such as the aberrant activation of the PI3K/AKT), some research discussed the lncRNA-mediated regulation of the mTOR pathway and the mechanisms through lncRNAs control mTOR signaling in TNBC. For example, LINC01133 was suggested to be downstream of the PI3K pathway and functioned as an upstream regulator of PI3K-AKT. Mechanically, it activated AKT in a PI3K-independent manner and exerted oncogenic activity in TNBC in a mTORC2-dependent pathway, in which LINC01133 induced the expression of PROTOR1/PRR5 (mTORC2 component) by coupling to a negative regulator of PRR5, hnRNPA2B1 [44]. A small-molecule Comp34 preferentially targeted TNBC and cancer stem cells and inhibited AKT1/mTOR expression by inhibition of lncRNA NUDT3-AS4, which sponged miR-99s to release the mRNA of AKT1/mTOR from miRNA-dependent decay [45]. VEGF-stimulated lncRNA PCAT6 was upregulated in TNBC tissues and cells, driving its proliferation, migration, invasion, and EMT process. PCAT6 further induced the vascular endothelial growth factor receptor 2 (VEGFR2)/AKT/mTOR pathway and angiogenesis, functioning as a sponge of miR-4726-5p and recruiting USP15 to regulate VEGFR2 [46]. LncRNA BDNF-AS induced endocrine resistance and malignant progression of BC, and it promoted ribonuclease/angiogenin inhibitor 1 (RNH1) protein degradation via tripartite motif containing 21 (TRIM21) and abolished RNH1-dependent mTOR mRNA decay, activating mTOR signaling [47]. Overexpressed lncRNA NAMPT-AS activated mTOR pathway via NAMPT-AS/POU class 2 homeobox 2 (POU2F2)/nicotinamide phosphoribosyltransferase (NAMPT) and NAMPT-AS/miR-548b-3p/NAMPT axes, promoting cell survival and invasiveness in TNBC [48]. Qiu et al. have described the relationship between AFAP1-AS1 and the mTOR pathway. Metformin exerted antitumor activities in part by regulating the AFAP1-AS1/miR-3163/secreted phosphoprotein 1 (SPP1) axis in lung adenocarcinoma, and miR-316 overexpression caused suppression of PI3K/AKT/mTOR signaling, which could be re-activated by SPP1 overexpression [49]. 

In this study, after analyzing the mRNA expression after AFAP1-AS1 silencing in both MDA-MB-231 and MDA-MB-468 cells, some genes were regulated in the same trend, such as SGK1, EIF4B, MAPKAP1, NEDD4L, SEH1L, SKP2. Q-RT-PCR and western blot assay confirmed the expression of these six genes. It seems reasonable to presume that AFAP1-AS1 could activate the mTOR pathway, leading to the expression change in several related genes, thereby contributing to tumorigenesis. However, the mechanism of how AFAP1-AS1 regulates the mTOR pathway still needs further exploration. Overall, the current study set out to explore the upstream mechanism of AFAP1-AS1 regarding TNBC and uncovered that AFAP1-AS1, activated by transcription factor SP1, could activate the mTOR pathway, consequently facilitating the progression of TNBC, which might be a therapeutic target for TNBC.

## 4. Materials and Methods

### 4.1. Cell Culture

MDA-MB-231 (ATCC^®^ HTB-26™, Stem Cell Bank, Chinese Academy of Sciences, Shanghai, China) and MDA-MB-468 (BNCC339862, BeNa Culture Collection, Beijing, China) cells were cultured in L15 medium containing 20% fetal bovine serum at 37 °C and 100% of constant incubator air temperature, respectively.

### 4.2. Transfection

For the knockdown of SP1 and YY1, we transfected 50 nM siRNAs against SP1 (si-SP1), YY1 (si-YY1) by lipofectamine 3000 (L3000015, ThermoFisher Scientific, Shanghai, China) in MDA-MB-231 and MDA-MB-468 cells (Table S1). SiRNAs that could not target them were the negative controls (si-NC). 

For overexpression of SP1, the pcDNA3.1 (vector) functioned as an empty vector to conduct the overexpressed plasmid (ov-SP1), and MDA-MB-231 and MDA-MB-468 cells were transfected with 2 μg ov-SP1 plasmid by lipofectamine 3000. 

For the knockdown of AFAP1-AS1, 2 μg sh-NC and sh-AFAP1-AS1 were transfected by lipofectamine 3000.

### 4.3. Quantitative Real-Time Polymerase Chain Reaction (Q-RT-PCR)

We extracted the total RNA from cells using the TRIzol reagent (Invitrogen, ThermoFisher Scientific, Shanghai, China). Moreover, we synthesized the cDNA by PrimeScript^TM^ RT Reagent Kit (RR037A, Takara, Beijing, China). We then conducted Q-RT-PCR by SYBR Premix Ex Taq^TM^ II (RR820A, Takara, Beijing, China). Table S1 shows the primers used in this study.

### 4.4. Western Blot

Proteins were isolated utilizing 10% SDS-PAGE and then transferred to PVDF membranes at 4 °C 200 mA for 1 h. The membrane was blocked for 0.5 h at room temperature and then incubated with primary antibodies, SP1 Antibody (21962-1-AP, Proteintech, San Diego, CA, USA), YY1 Antibody (66281-1-Ig, Proteintech, San Diego, CA, USA), EIF4B Antibody (YN5712, 1:1000, Immunoway, Plano, TX, USA), MAPKAP1 Antibody (YT7258, 1:500, Immunoway, Plano, TX, USA), SGK1 Antibody (YT4268, 1:500, Immunoway, Plano, TX, USA), SEH1L Antibody (ab218531, 1:1000, Abcam, Shanghai, China), SKP2 Antibody (YT4311, 1:500, Immunoway, Plano, TX, USA), NEDD4L Antibody (YN3050, 1:500, Immunoway, Plano, TX, USA), GAPDH Antibody (60004-1-Ig, Proteintech, San Diego, CA, USA), followed by incubating with HRP secondary antibody for 1 h. Finally, the proteins were measured by the Super ECL Plus Kit (PK10002, Wuhan Sanying, Wuhan, China).

### 4.5. Chromatin Immunoprecipitation Assay (ChIP)

ChIP assay was performed using Pierce Magnetic ChIP Kit (26157, ThermoFisher Scientific, Shanghai, China). Immunoprecipitation was incubated with anti-SP1 (ab231778, Abcam, Shanghai, China) or anti-IgG (negative control). Finally, Q-RT-PCR was used to detect precipitated chromatin DNA. Table S1 shows the primers used in this study.

### 4.6. Dual Luciferase Assay

LncRNA-AFAP1-AS1 wild type plasmid (P3 region) and LncRNA-AFAP1-AS1 mutant plasmid (P3 region) were co-transfected with SP1 overexpression plasmid (ov-SP1) and control vector (pcDNA3.1) into MDA-MB-231 cells. The relative luciferase activity was measured by Dual-Luciferase^®^ Reporter Assay System (E1910, Promega, Madison, WI, USA).

### 4.7. Cell Counting Kit-8 (CCK-8)

The 5 × 10^4^ cells from each group were inoculated in a 96-well plate. At 0 h, 24 h, 48 h, and 72 h, we added 10 μL CCK-8 detection solution to each well and incubated it for 4 h. The absorbance at 450 nm was measured by BIOBASE-EL10A (OLABO, Shandong, China).

### 4.8. Colony Formation Assay

The cells of each group were digested, counted, and diluted. The 500 cells were inoculated in a 12-well plate and cultured in a cell incubator for 3 weeks. Then, we discarded the culture medium, washed it twice with PBS, and added 4% paraformaldehyde to fix it for 15 min. After removing paraformaldehyde, we added 1 mL Giemsa for 15 min, washed it with PBS, and dried and photographed the plate.

### 4.9. Wound Healing Assay

When the cells were full in the 12-well plates, we used a micropipette tip to draw lines along the central axis of the wells and then added the culture medium to the wells after PBS washing. We observed the migration distance of cells and calculated the cell migration rate at 0 h and 48 h. 

### 4.10. Transwell Assay

We spread 100 µL of Matrigel on the bottom of the Transwell chamber (Millicell, Millipore, Shanghai, China) and put it in the incubator for 2–3 h until it ultimately solidified. We then added 600 µL of complete medium to the lower layer of the Transwell chamber. We resuspended 1 × 10^5^ cells in 100 µL of serum-free medium and transferred it to Matrigel’s surface. After incubation of 24 h (MDA-MB-231 cells) and 48 h (MDA-MB-468 cells), we discarded the old culture medium and washed it twice with PBS. We wiped the cells above the chamber with a cotton swab, and fixed it with ice-cold 4% paraformaldehyde for 30 min, stained it with Giemsa for 15 min, washed the lower part of the chamber with PBS, and took a photomicrograph of the Transwell chambers.

### 4.11. In Vivo Tumor Formation Model

The protocol and procedures employed were ethically reviewed and approved by the Animal Ethics Committee of Peking Union Medical College Hospital.

The 24 SPF BALB/C strain nude mice (Speyford, Beijing, China) used in the experiment were 1–5 weeks old, female, healthy, and mature, with an average 18 ± 2 g weight. After 1 week of adaptive feeding, the nude mice were randomly divided into 4 groups. The specific groups were vector, ov-SP1, vector + sh-AFAP1-AS1, and ov-SP1 + sh-AFAP1-AS1.

Cells in each group were digested with trypsin, washed twice with PBS, and diluted to 10^7^/mL with serum-free medium. Under aseptic conditions, the nude mice were inoculated subcutaneously under the armpit with 0.1 mL cell suspension and kept for 30 days.

The tumor was measured every week by Vernier caliper, and the tumor volume was calculated according to the formula V = 1/2AB^2^ (A = the longest diameter of the tumor body; B = the shortest longitude of the tumor body). Moreover, the tumor growth curve of each group was drawn (*Y* axis = the tumor volume; *X* axis= the number of growth weeks).

### 4.12. Immunohistochemical Staining

The extracted mouse tumor tissues were weighted and then fixed with 4% paraformaldehyde, embedded in paraffin, and sliced into sections. The paraffin in the sections was removed with xylene, and the sections were then rehydrated with gradient ethanol and distilled water. After PBS washing 3 times, 3% H_2_O_2_ was used to block and inactivate endogenous peroxidase at room temperature for 10 min, followed by PBS washing 3 times. The sections were then heated in 0.01 M citric acid buffer (PH 6.0) (95 °C for 20 min), cooled naturally for more than 20 min, washed with PBS, blocked by 20% goat serum for 20 min, and probed with 50 μL primary antibody at 4 °C overnight, followed by 37 °C reheating for 45 min. After that, EnVision reagent was dropped at room temperature for 30 min, followed by DAB development for 10 min. The sections were then counterstained with hematoxylin for 2 min and differentiated in hydrochloric acid. After rinsing for 15 min, the sections were dehydrated, cleared, and sealed for microscopic examination.

### 4.13. Fluorescence In Situ Hybridization (FISH)

Paraformaldehyde (4%) fixed the MDA-MB-231 and MDA-MB-468 cells, followed by the treatment of PBS three times (pH 7.4). Then, cells were digested by Protease K (20 μg/mL) and cultured with a specific probe overnight. After the hybridization, the slices were dripped with DAPI dye, incubated in the dark for 8 min, and then dripped with an anti-fluorescence quenching sealing agent after washing. A fluorescence microscope (Leica) captured all fluorescence images. Table S1 shows the sequence for the AFAP1-AS1 probe.

### 4.14. GeneChip^®^ PrimeView™ Human Gene Expression Array

The GeneChip^®^ PrimeView™ Human Gene Expression Array Kit (Affymetrix, ThermoFisher) uses a probe set to realize expression spectrum analysis, and the sequence used in array design is from RefSeq V36, UniGene 219 and full-length human mRNA in GenBank ™. The experiment was conducted in strict accordance with the instructions of ThermoFisher. Total RNA was extracted from MDA-MB-231 and MDA-MB-468 cells after the knockdown of lncRNA AFAP1-AS1, followed by RNA quality control. cDNA was synthesized from total RNA after adding PolyA control, followed by biotin labeling, purification, and quantification of cRNA. The fragment labeled cDNA was added to the gene chip, and we conducted the hybridization in the GeneChip™ Hybridization Oven 645 (ThermoFisher) and dye in GeneChip™ Fluidics Station 450 (ThermoFisher) according to the corresponding protocol. GeneChip™ 3000 7G (ThermoFisher) was used to capture the fluorescence signal, and GCOS was used to convert the signal to generate the CEL file.

### 4.15. Statistical Analysis

Prism statistical software implemented the statistical analyses. Measurement data are expressed as mean ± standard deviation (mean ± SD). Unpaired Student’s *t*-tests were used for the comparison between the two groups. One-way ANOVA was used for the statistical analyses between three or more groups. Two-way ANOVA was used for the statistical analyses between three or more groups in CCK-8 and in vivo tumor growth experiments. * represented *p* < 0.05; ** represented *p* < 0.01; *** represented *p* < 0.005; ns represented not significant.

## Data Availability

All data are available, and the correspondent can be contacted if requested.

## Supplementary Materials

The following supporting information can be downloaded at https://www.mdpi.com/article/10.3390/ijms241713401/s1.
