# Supplementary material for: SP1-Induced Upregulation of LncRNA AFAP1-AS1 Promotes Tumor Progression in Triple-Negative Breast Cancer by Regulating mTOR Pathway
Source: Int J Mol Sci. 2023 Aug 29;24(17):13401. doi: 10.3390/ijms241713401 (PMC10563082; doi:10.3390/ijms241713401)
Supplement: Supplementary file 1 [file ijms-24-13401-s001.zip › ijms-2547637-supplementary.pdf]

Table S1. Primers or sequences used in the study.

siRNAs	
si-SP1	GUGCAAACCAACAGAUUAUTT
	AUAAUCUGUUGGUUUGCACTT
si-YY1	CAUGCUAAGGCCAAAAACATT
	UGUUUUUGGCCUUAGCAUGTT
Q-RT-PCR primers	
lncRNA-AFAP1-AS1	FW: 5'-AATGGTGGTAGGAGGGAGGA-3'
	RV: 5'-CACACAGGGGAATGAAGAGG-3'
GAPDH	FW: 5'-ATGACATCAAGAAGGTGGTGAAGCAGG-3'
	RV: 5'-GCGTCAAAGGTGGAGGAGTGGGT-3'
YY1	FW: 5'-TGGCAGAATTTGCTAGAATGAAGC-3'
	RV: 5'-TGGCCGAGTTATCCCTGAAC-3'
SP1	FW: 5'-TTGCTGCTATGCCAAACCTA-3'
	RV: 5'-CCTGAGAGCTGGGAGTCAAG-3'
EIF4B	FW: 5'-CTTCCCAAATCGCCACCC-3'
	RV: 5'-CGAGCCCTCCAGTCTGTATCT-3'
MAPKAP1	FW: 5'-AACAGTTCAAGGGCAGACGG-3'
	RV: 5'-CAGCAAGGTCACAGGCACA-3'
SGK1	FW: 5'-ACCATCTCCAGAGGGAACGC-3'
	RV: 5'-GCAGGCCATACAGCATCTCATAC-3'
SEH1L	FW: 5'-TCAGCAGGGAACCTCAAATC-3'
	RV: 5'-GGCATAACTGGACCATCTCG-3'
SKP2	FW: 5'-GCTCAACTACCTCCAACACCT-3'
	RV: 5'-TCTGGCACGATTCCAAAA-3'
NEDD4L	FW: 5'-CGACCCTATACATTTAAGGACTTTC-3'
	RV: 5'-TGGTGCTGAGAAGCCGAGT-3'
CHIP primers	
P1 primer for ChIP	5'-GAGACAGCGTCCATTCACTCCC-3'
	5'-ACAAAGATTTCCTCCTAACCTTCC-3'
P2 primer for ChIP	5'-GACCCACGTGACACGCC-3'
	5'-CCCACGACACCCGTAAGA-3'
P3 primer for ChIP	5'-GTACGTGCCCAATACTTGACG-3'
	5'-GGCTTTGTTCCAGGTGCTT-3'
P4 primer for ChIP	5'-AGCCAGTGCTAGACAGCAACAA-3'
	5'-AGCCAAACCATATCACCTCCTT-3'
P5 primer for ChIP	5'-GCACCTGTGCAGGAAGAG-3'
	5'-TGAGATTTGGATGGGGATAC-3'
P6 primer for ChIP	5'-CATCCAAATCTCAGCTTGAATT-3'
	5'-CCTTGATAAACCCTCCAGGTA-3'
FISH probes	
AFAP1-AS1	AAGGGGCTCAAAGATACACCAAAGT

	ATGTTGGATGTCTAATCAGGGAGCC<br>CACCCTCTTCCCTTCCCTGTAGTAG<br>CAGAGAAGCATTAAGACATGAGACACT<br>TGAGAAAAGAATCACCAAGAGAGTAAG

Table S2. The potential binding sites of Sp1 towards lncRNA AFAP1-AS1 promoter regions (hg38_knownGene_ENST00000608442.2, range = chr4:7752077-7779028) by JASPAR with relative profile score threshold of 80 %.

Matrix ID	*Name*	*Score*	*Relative score*	*Start*	*End*	*Strand*	*Predicted sequence*
MA0079.1	MA0079.1.SP1	9.304687	0.911425279	1175	1184	+	CGGGCGTGGT
MA0079.3	MA0079.3.SP1	9.048192	0.894977296	790	800	+	TTTCCTCCCCA
MA0079.1	MA0079.1.SP1	8.938865	0.89866482	1792	1801	+	GAGGCAGGGA
MA0079.5	MA0079.5.SP1	8.318838	0.863441621	1878	1886	+	TGGGAGGGA
MA0079.1	MA0079.1.SP1	8.311052	0.876765707	1037	1046	+	TGGGCCGGGT
MA0079.3	MA0079.3.SP1	8.113572	0.883218707	766	776	+	GCCCATCCCCC
MA0079.5	MA0079.5.SP1	8.020701	0.858261002	1245	1253	+	GAGGCGGAG
MA0079.1	MA0079.1.SP1	7.197659	0.837928744	1877	1886	+	GTGGGAGGGA
MA0079.1	MA0079.1.SP1	6.957125	0.829538534	1911	1920	+	GGGGGCTGGT
MA0079.1	MA0079.1.SP1	6.7868495	0.823599042	273	282	+	TGGGGATGGA
MA0079.5	MA0079.5.SP1	6.593582	0.833462442	1798	1806	+	GGGAAGGAG
MA0079.3	MA0079.3.SP1	6.4261227	0.861988661	1509	1519	+	CGCCTGCCCCC
MA0079.1	MA0079.1.SP1	6.1590366	0.801699912	160	169	+	TGGGGAAGGT
MA0079.5	MA0079.5.SP1	5.623053	0.816597892	274	282	+	GGGGATGGA
MA0079.4	MA0079.4.SP1	5.5256863	0.845683731	758	772	+	CGTGACACGCCCATC
MA0079.5	MA0079.5.SP1	5.255307	0.810207699	1037	1045	+	TGGGCCGGG
MA0079.3	MA0079.3.SP1	5.1665006	0.846141173	100	110	+	TTCACTCCCCC
MA0079.3	MA0079.3.SP1	2.6205626	0.814110358	765	775	+	CGCCCATCCCC
MA0079.3	MA0079.3.SP1	2.4257746	0.811659703	1918	1928	+	GGTCTTCCCCG
MA0079.3	MA0079.3.SP1	2.1372697	0.808029981	770	780	+	ATCCCCCCCAT
MA0079.4	MA0079.4.SP1	0.9273069	0.804953733	1690	1704	+	CAGAACACGCACCTG
MA0079.4	MA0079.4.SP1	0.9273069	0.804953733	1722	1736	+	CAGAACACGCACCTG
MA0079.4	MA0079.4.SP1	0.7430556	0.803321733	763	777	+	CACGCCCATCCCCCC

Table S3. The intersection of down-regulated genes in MDA-MB-231 and MDA-MB-468 cells after knockdown of lncRNA AFAP1-AS1, and the relative KEGG pathways of these 17 down-regulated genes which appeared more than twice.

Overlapped down-regulated genes	*Relative KEGG pathways*
ARSD	hsa00500: Starch and sucrose metabolism
UBE2I	hsa04120: Ubiquitin mediated proteolysis
KLF6	hsa04960: Aldosterone-regulated sodium reabsorption
COMT	hsa04121: Ubiquitin system BR
DUSP1	hsa03019: Messenger RNA biogenesis BR
NANOS1	**hsa04150: mTOR signaling pathway**
GYG2	hsa04151: PI3K-Akt signaling pathway
C6orf120	hsa04990: Domain-containing proteins not elsewhere
NEDD4L	classified BR
ENPP1	hsa03000: Transcription factors BR
SGK1	
FAM172A	
MATN2	
EIF4B	
LMO4	
FOSB	
CPA4	

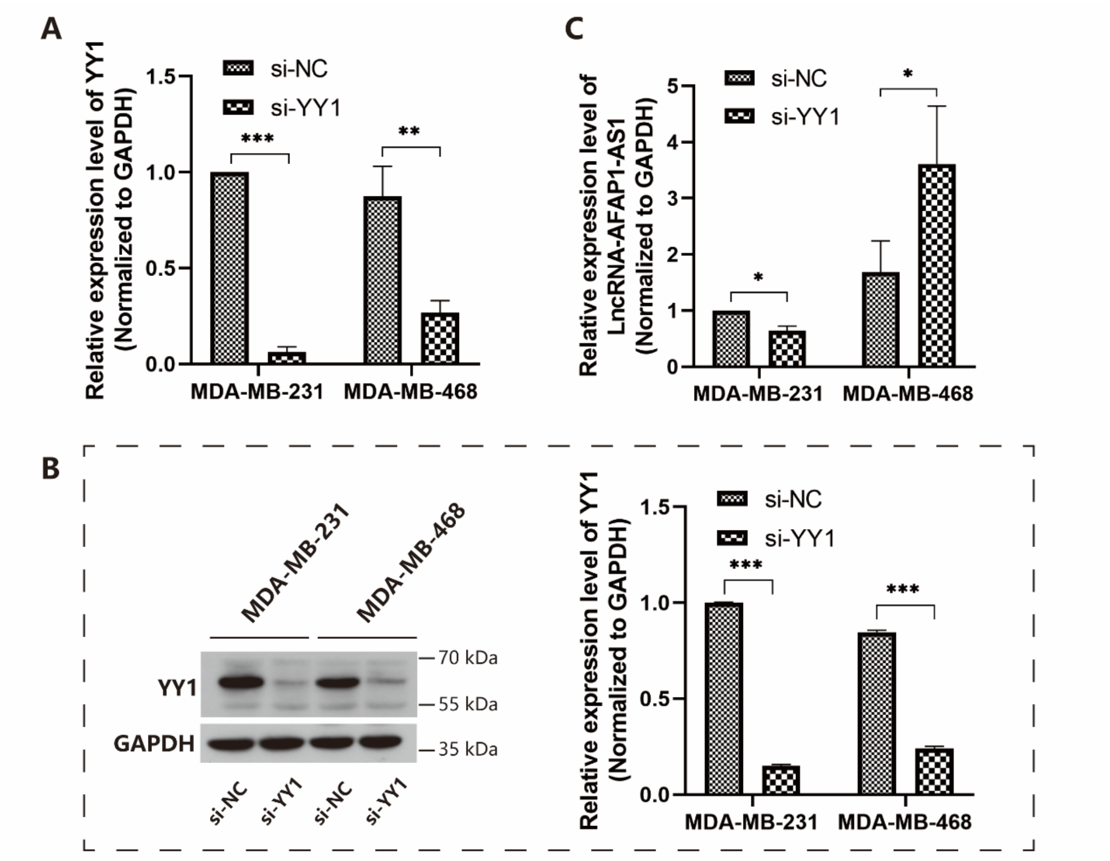

Figure S1 The regulation of YY1 towards the expression of lncRNA AFAP1-AS1. (A) Q-RT-PCR and (B) western blot assays confirmed the silencing efficiency of YY1 by siRNA; (C) lncRNA AFAP1-AS1 expression was decreased in MDA-MB-231 cell after YY1 silencing and increased in MDA-MB-468 cell. Unpaired student's t-tests were used for the statistical analyses. *p < 0.05; **p < 0.01; ***p < 0.005; ns, no significant.
